# The complete chloroplast genome of *Asparagus densiflorus* (Kunth) Jessop L.

**DOI:** 10.1080/23802359.2022.2068975

**Published:** 2022-04-29

**Authors:** Rendian Zhang, Wentao Sheng

**Affiliations:** Department of Biological Technology, Nanchang Normal University, Nanchang, Jiangxi, People’s Republic of China

**Keywords:** Chloroplast genome, *Asparagus densiflorus*, maximum-likelihood method, phylogenetic tree analysis

## Abstract

*Asparagus densiflorus* (Kunth) Jessop L. is a horticultural plant widely cultivated in China. Herein, we reported the complete chloroplast (cp) genome of *A. densiflorus* from the genus *Asparagus*. The entire cp genome of *A. densiflorus* was 157,141 bp in length with one large single-copy region of 91,255 bp and one small single-copy region of 20,355 bp, separated by a pair of inverted-repeat regions of 45,531 bp. The GC content is 36.46% in this cp genome. A total of 134 genes were annotated including 90 protein-coding genes, 36 tRNA genes, and 8 rRNA genes. The maximum-likelihood phylogenetic analysis showed that *A. densiflorus* was the most closely related to *Asparagus cochinchinensis*.

The genus *Asparagus* is a complex group widely distributed in the old world with high economic value, including perennial herbs, shrubs, and vines (Kubota et al. 2012). *Asparagus densiflorus* (Kunth) Jessop, Bothalia.9:65.1966. is an important horticultural plant in this genus, with high economic and ornamental value. It is originated in Africa, but is widely cultivated in China at present. There are more than 200 species in this genus, many of which are similar in morphology. And *A. densiflorus* is difficult to distinguish from other *Asparagus* plants in morphology (Fukuda et al. 2005). It is reported that DNA barcode, a special DNA sequence used in species identification, has become a focus in biological taxonomy and species discrimination research in recent years (Kang 2021). But there are few DNA sequences are released for *A. densiflorus* in GenBank. Herein, we sequenced the complete chloroplast (cp) genome sequence of *A. densiflorus* to serve as a genetic resource for further studies on the taxonomy and species identification of *Asparagus* plants, and to get a better understanding of phylogenetic relationship in the genus.

The fresh leaves of *A. densiflorus* were collected from Nanchang, China. The samples were stored in Nanchang Normal University (Nanchang, Jiangxi, China, 115°27′E, 28°09′N). A specimen was deposited at Zoological and Botanical Specimen Museum of the College of Life Science (http://swx.ncnu.edu.cn/, the contact person is Wentao Sheng and the email is shengwentao2003@163.com) under the voucher number NCNU-B-1025. A CTAB protocol was used to isolate total genomic DNA (Li et al. 2013). Sequencing libraries were generated using a TruSeq DNA Sample Preparation Kit (Illumina, USA) and a Template Prep Kit (Pacific Biosciences, USA). Genome sequencing was performed using Illumina NovaSeq platform. A total of 18,275,612 high-quality reads were obtained, and 16,892,743 clean reads were assembled using SPAdes (Bankevich et al. 2012) and A5-miseq (Coil et al. 2015) to construct high quality scaffolds and contigs. The cp splicing results were obtained using these software packages, and the reference genome (*A. officinalis* L.: NC_034777.1) was analyzed using Mummerv 3.1 software (Kurtz et al. 2004) to determine the positional relationship and to fill the gaps between contigs. Results were corrected using Pilonv1.18 software (Walker et al. 2014) to obtain the final cp genome. Its functional annotation of gene sequence was performed using the online program GeSeq (https://chlorobox.mpimp-golm.mpg.de/geseq.html) in the entire assembled cp genome.

The whole cp genome of *A. densiflorus* was a circular structure with 157,141 bp, including one large single copy (LSC) region (91,255 bp), one small single copy (SSC) region (20,355 bp), and a pair of IRs regions (45,531 bp). And the GenBank accession number is MT740250.1. The overall GC content of the genome, the LSC, SSC, and IR regions were 36.46%, 35.7%, 31.8%, and 43.1%, respectively. The cp genome annotation contained a total of 134 genes, including 90 protein-coding genes, 36 tRNA genes, and 8 rRNA genes. In addition, 6 protein-coding genes (*rp12*, *rp123*, *ycf2*, *ndhB*, *rps7* and *rps12*), 7 tRNA genes (*trnN-GUU*, *trnR-ACG*, *trnA-UGC*, *trnI-GAU*, *trnL-CAA*, *trnV-GAC* and *trnI-CAU*), and all of rRNA genes (*rrn4.5*, *rrn5*, *rrn16*, *rrn23*) were duplicated in the IR regions. In total, 10 genes had a single intron, of which 6 genes (*rpoC1*, *petB*, *rpl16*, *atpF*, *petD* and *rps16*) were protein-coding genes, and 4 genes (*trnI-GAU*, *trnA-UGC, trnK-UUU,* and *trnL-UAA)* were tRNA genes. Moreover, 5 genes (*rps12*, *rp12*, *ycf3*, *ndhB* and *clpP*) contained two introns.

To confirm the phylogenetic relationship of *A. densiflorus* with other plant species in Asparagaceae, the complete cp genome sequences of 18 species from this family in combination with *Allium cernuum* (NC_057572.1) as the out-group were obtained from GenBank, and then were aligned by the MAFFT v7.388 (Katoh and Standley 2013) and trimmed properly by trimAl (Capella-Gutierrez et al. 2009). Based on the single copy core gene identified by corepan analysis, multiple protein sequences were compared by MUSCLE software (https://www.swmath.org/software/13193) and transformed into coding sequences results. The evolutionary tree was reconstructed by maximum-likelihood model of TreeSoft (http://treesoft.sourceforge.net/treebest.shtml). The phylogenetic tree indicated that *A. densiflorus* is a sister group to the clade including to *Asparagus filicinus* to *Asparagus setaceus*, and was the most closely related to *Asparagus cochinchinensis* (Figure 1). Therefore, this complete reported cp genome will provide useful information for molecular identification, genetic diversity analysis, and resource exploitation of *A. densiflorus*.

**Figure 1.**
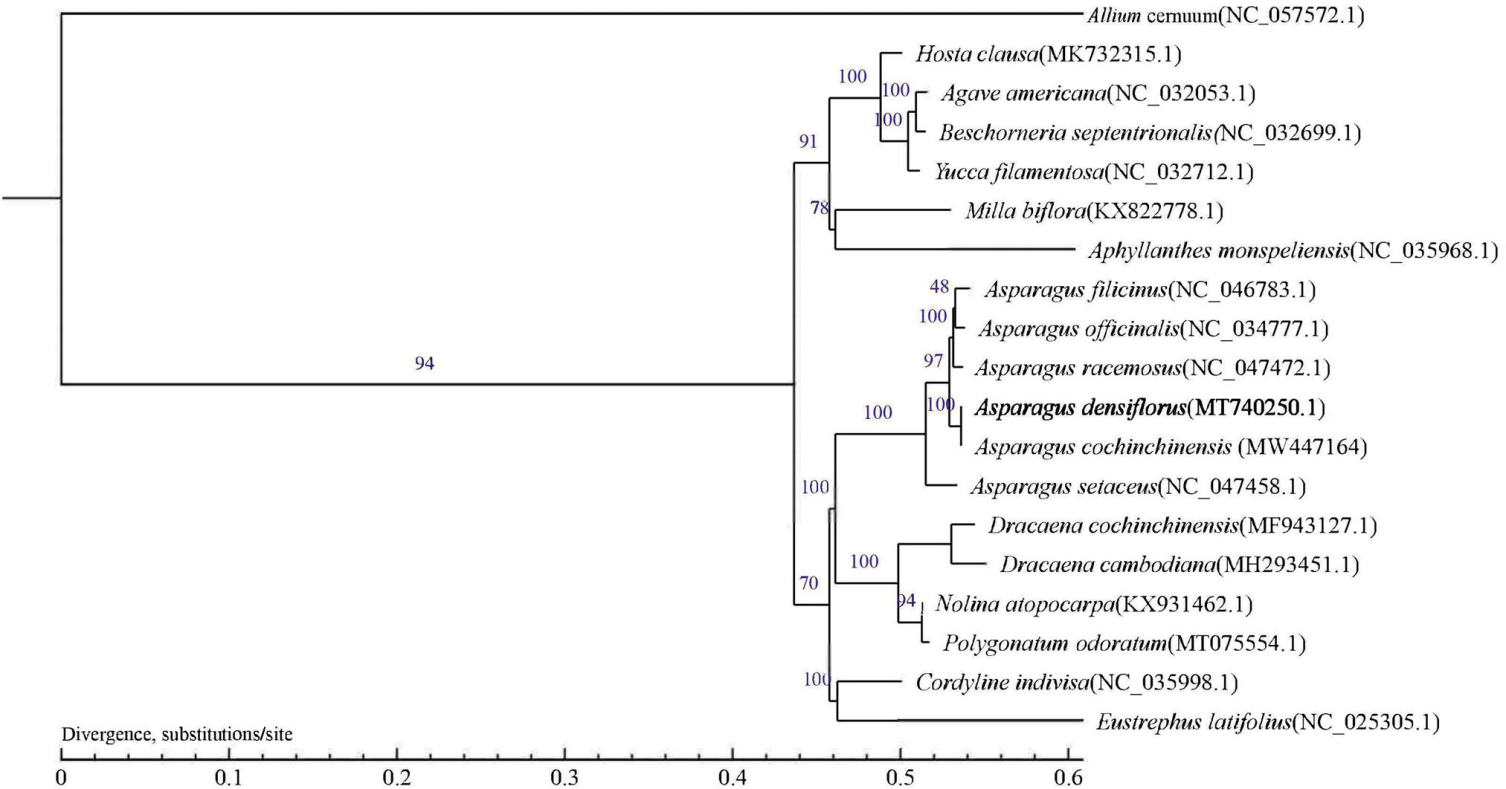
The maximum-likelihood (ML) phylogenetic tree was reconstructed based on the single copy core gene with the cp genomes of 18 species from Asparagaceae, along with *Allium cernuum* (NC_057572.1) as the out-group. Numbers above the lines represented ML bootstrap values.

## Author contributions

Wentao Sheng was involved in the conception and design, or analysis and interpretation of the data; Rendian Zhang was involved in the drafting of the paper, revising it critically for intellectual content and the final approval of the version to be published; and the authors agrees to be accountable for all aspects of the work. No potential conflict of interest was reported by the authors.

## Ethics statement

The collection of specimen conformed to the requirement of International ethics, which did not cause damage to the local environment. The process and purpose of this experimental research were in line with the rules and regulations of our institute. There are no ethical issues and other conflicts of interest in this study.

## Data Availability

The genome sequence data that support the findings of this study are openly available in GenBank of NCBI at [https://www.ncbi.nlm.nih.gov] (https://www.ncbi.nlm.nih.gov/) under the accession number MT712150.1. The associated BioProject, SRA, and Bio-Sample numbers are PRJNA752952, SRR15371401, and SAMN20668209, respectively.
